# Milk composition, traditional processing, marketing, and consumption among smallholder dairy farmers in selected towns of Jimma Zone, Oromia Regional State, Ethiopia

**DOI:** 10.1002/fsn3.2884

**Published:** 2022-04-16

**Authors:** Belay Duguma

**Affiliations:** ^1^ College of Agriculture and Veterinary Medicine Department of Animal Science Jimma University Jimma Ethiopia

**Keywords:** awareness of zoonotic diseases, milk composition, milk consumption, milk marketing, milk processing

## Abstract

In Ethiopia, dairy products remain the most important animal source of foods including in the current study area. However, poor milk quality is frequently told constraint, and this affects smallholder dairy development. This study aimed to assess raw cow milk composition, processing, consumption, and marketing among smallholder dairy producers in selected towns of Jimma Zone, Ethiopia. Primary data were collected by surveying 52 randomly selected dairy cattle keeping households using a structured questionnaire and analysis of raw milk chemical composition. The results revealed that the average milk production was 5.98 ± 1.01 liters/household/day, of which the majority (62.9%) was marketed. About 22.7% of households reported processing milk into butter, cottage cheese, ghee, and fermented milk at household level mainly for domestic use. A combination of fresh whole milk, fermented milk, butter, cottage cheese, and ghee (51.7%) were the main dairy products consumed by respondents. About 72.2% and 6.1% of households consumed boiled and raw milk, respectively, while 100% respondents reported consuming raw fermented whole milk. Milk was channeled to consumers and retailers through informal marketing system (100%). The awareness of respondents about zoonotic diseases was found to be very low. The mean fat, lactose, and protein content of raw cow milk were significantly (*p* < .05) affected by breed. It is recommended that consumption of raw milk and fermented milk could pose a public health risk to consumers, implying the need for urgent farmers’ awareness creation to boil milk before consumption for prevention and control of zoonotic diseases.

## Graphical abstract

The fat, lactose, and protein content of raw milk varied significantly (*p* < .05) between crossbred and indigenous cows. Raw whole milk was processed into fermented milk, butter, cottage cheese, and ghee at household level. Raw milk was channeled to consumers and retailers through informal marketing system. 72.2% household boiled whole raw milk before consumption while 6.1% and 100% of respondents consumed raw whole milk and raw fermented whole milk, respectively.

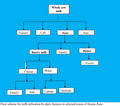

## 1 INTRODUCTION

Agriculture is a lifeblood and economic backbone of Ethiopia, and livestock is an integral part of the agriculture. Ethiopia has the largest livestock population in Africa, with estimated 65.35 million head of cattle, 39.89 million sheep, 50.50 million goats, 2.11 million horses, 8.98 million donkeys, 0.38 million mules, and about 7.70 million camels (CSA, 2020). Livestock farming is an important and integral component of the agriculture and rural livelihood in the country contributing about 17%–25.3% of the national gross domestic product (GDP) and 39%–49% of agricultural GDP and over 50% of household income (Shapiro et al., 2017), and 12%–15% of the export earnings, and provide employment for about 60%–70% of the population (Tegegne et al., 2013). The GDP of livestock‐related activities was valued at birr 59 billion (Metaferia et al., 2011). Livestock also contributes products such as draught power, meat, milk, eggs, hides and skins, manure for fuel and fertilizer, and surplus products are marketed earning farmers income and therefore contributing to household food security.

As indicated above, Ethiopia also has the largest cattle population in Africa, estimated to be around 65.35 million head, of which about 97.76% of the total cattle in the country are local breeds (nondescriptive type, which do not belong to any specific breed). The remaining are hybrid and exotic breeds that accounted for about 1.91% and 0.32%, respectively. Dairy cows are estimated to be around 7.15 million and milking cows are about 12.57 million heads (CSA, 2020). Despite the large cattle population, milk productivity is very low, and the annual per capita milk consumption is very low, estimated at about 20 L, though rising consumption levels in Addis Ababa (capital city of Ethiopia) have brought it to about 40 L (Barry et al., 2017). This is extremely less than 200 L of per capita consumption recommended by the World Health Organization (WHO).

Population growth, increasing urbanization, rising incomes, and preferences for animal sources of food are predicted to double the demand for, and production of, livestock and livestock products in developing countries over the next 20 years (Delgado et al., 1999). Projections indicate a large increase in demand for dairy products, particularly in developing countries including sub‐Saharan Africa (Delgado et al., 1999). Per capita food consumption of animal products continues to increase both in developing and developed countries, as well as in countries in transition, as a result of increasing average per capita real income (Popkin & Du, 2003; Speedy, 2003).

Eastern Africa is the leading dairy producer in Africa, and approximately 68% of dairy products of the continent come from Ethiopia, Kenya, and Tanzania (Bingi & Tondel, 2015). It is estimated that the dairy sector contributed 9%–14% of East Africa's agricultural gross development product (Lukuyu et al., 2019).

In Ethiopia, dairy production is one of the oldest livestock subsector and is characterized almost all by rural smallholder dairy production using indigenous cattle, and dairy production using dairy breeds is still in the initial stages. The smallholder farmers are the main and the most important producers of milk with about 98% of the country's milk supply. The national average daily milk yield and lactation period of local cow are 1.37 L and 6 months, respectively (CSA, 2020). This shows low productivity per head of local cow attributed to feed shortage in terms of quality and quantity, high disease and parasite incidences, and low genetic potential of the local breed, among others. Smallholder dairy sector contributes to approximately 16.5% of the national GDP, 35.6% of the agricultural GDP, and 15% of export earnings and 30% of agricultural employment (Behnke, 2010; Metaferia et al., 2011). Ethiopia produces three billion liters of milk per year using local cow (CSA, 2020).

In Ethiopia, cattle are the major sources of milk contributing approximately 83% of the total milk production (LDMPS, 2007). As dairying plays significant role in the lives of the urban and peri‐urban resource poor households (Yitaye et al., 2007), promotion of the dairy sector in Ethiopia can therefore contribute significantly to poverty alleviation as well as availability of food and income generation. Dairy products form part of the diet of many Ethiopians. At the rural dairy farming system in Ethiopia, 68% of the total milk produced is used for human consumption in different forms (Getachew & Gashaw, 2001). The milk sector in Ethiopia is expected to continue growing in the future given the large potential for milk development in the country, the expected growth in income, increased urbanization, and improved policy environment (Mohamed et al., 2004). In Ethiopia, the urban population increases at a rate of 4.4% per annum. Therefore, increase in population and consumer income in the future is expected to increase the consumption of dairy products (Mohamed et al., 2004).

Milk is very important for nutrition of the young, and milk‐borne biologically active compounds such as casein and whey proteins have been found to be increasingly important for physiological and biochemical functions that have crucial impacts on human metabolism and health (Gobbetti et al., 2002). Park (2009) also indicated that these compounds have been found to be useful in guarding humans against pathogens and illnesses. Although milk has a high nutritional value (Gaucheron, 2011), it constitutes a good growth medium for bacteria, of which some are pathogenic to humans (Jayarao & Henning, 2001). Raw milk can be contaminated with pathogens even when sourced from clinically healthy animals (Soboleva, 2014). The unique composition and properties make milk an excellent medium for bacterial growth and a source of bacterial infection (Claeys et al., 2013). Milk‐borne pathogenic bacteria pose a serious threat to human health and constitute about 90% of all dairy‐related diseases (Ryser, 1998). *Staphylococcus aureus*, *Salmonella* spp., *Listeria monocytogenes*, *Escherichia coli* O157:H7, and *Campylobacter* are the main microbiological hazards associated with raw milk consumption (Claeys et al., 2013).

Milk is a complex mixture of compounds, that is, water, fat, protein, lactose, enzymes, minerals, organic acids, and vitamins (Schwendel et al., 2017). Milk composition is influenced by factors which are specific to a cow and its environment. These factors are breed, age, health status, stage of lactation, diet, the intensity of management, milking interval, and ambient environmental temperature and seasonality, which influence feed availability (Chen et al., 2014; Schwendel et al., 2017). Milk composition determines the economic feasibility of processing (i.e., the yield of butter, or cheese obtained per kg of milk) and affects the quality of dairy products (Chen et al., 2014). Low protein percentage has been reported in a handful of studies investigating milk composition in Kenya (Kabui et al., 2015; Ondieki et al., 2017). Moreover, milk quality and safety are important consumer requirements (Malek dos Reis et al., 2013). Milk composition (fat, protein, and lactose) could be used as a diagnostic tool for the herd management, in particular the health of lactating animals (Forsbäck et al., 2010).

Milk marketing study is essential to provide vital and valid information on the operation and efficiency of milk product marketing system for effective research, planning, and policy formulation (Adebabay, 2009). Milk products in Ethiopia are marketed through both formal and informal value chains. According to Yilma et al. (2011), around 95% of the milk marketed in Ethiopia at national level was reported to be channeled through informal outlets which are characterized by direct delivery of fresh milk to immediate neighborhood consumers or catering service providers such as cafes, hotels, and restaurants without any quality control. From the point of view of policy makers, development agents, researchers, and private investors, information about the potential of urban milk production, processing, and marketing is limited.

Tegegne et al. (2013) indicated that the primary constraints under milk marketing and consumption included poorly understood structure and performance of the informal private sector, lack of reliable information on demand patterns, including product differentiation and changes in dairy consumption habit with urbanization, limited market information on input (e.g., feeds), and output markets. Also, concerns over public health hazards of marketed raw milk are associated with increased informal marketing, particularly brucellosis, zoonotic tuberculosis, and low standards of milk hygiene.

In Ethiopia, the vast majority of milk produced in rural areas are processed at household level into milk products such as *Ergo* (Ethiopian naturally fermented milk), butter, ghee, and *Ayib* (Ethiopian cottage cheese) using traditional technologies and are marketed through informal channel (Muriuki & Thorpe, 2008).

In Ethiopia, the government has developed a strategy aimed at increasing the development of dairy production in order to satisfy the increasing demand for milk and milk products in urban areas, such as major and secondary cities, and small towns to alleviate poverty among smallholder dairy producers. As a result, the number of urban and peri‐urban dairy farming was increasing in the recent decades and gaining importance to benefit from dairy development as a source of family food, income, and employment opportunity.

To this end, the need to understand milk composition, processing, consumption, and marketing, as well as farmers’ awareness of cattle‐ and milk‐borne zoonotic diseases under smallholder urban dairy farming condition, is important. District towns included in the current study are the high potential areas for milk production in Jimma zone. However, composition of milk is one of the major constraints among consumers; generally, lack of attention of policy planners and actual information on its functioning is limited. Therefore, understanding of milk composition, traditional processing, marketing, consumption, and farmers’ awareness of cattle‐ and milk‐borne zoonotic diseases would be useful to develop policies, development strategies, and business development services for the efficient value chain in smallholder milk marketing. The aim of this study was to assess raw cow milk composition, processing, consumption, and farmers’ awareness of cattle‐ and milk‐borne zoonotic diseases among smallholder dairy farmers in selected towns of Jimma Zone, Oromia Regional State, Ethiopia.

## 2 MATERIALS AND METHODS

### 2.1 Study areas

The present survey was conducted in the capital towns (namely Agaro, Seka, Sheki, Serbo, and Yebu) of the five districts of the Jimma zone, Oromia Regional State, Ethiopia. Jimma zone is 352 km southwest of Addis Ababa, the capital of Ethiopia. The geographical locations of the towns are as follows: Agaro, 7°40′N–8°04′N, 36°17′E–36°46′E; Seka, 7°17′N–7°44′N, 36°17′–36°42′E; Sheki, 7°13′N–8°39′N, 36°43′E–37°12′E; Serbo, 7°35′N–8°00′N, 36°46′E–37°14′E; and Yebu, 7°38′N–7°54′N, 36°38′E–36°53′E. The altitude of the areas ranges from 880 to 2660 m above sea level. The Agaro, Seka, Sheki, Serbo, and Yebu towns are located ~45, 18, 23, 23, and 22 km from the Jimma town, capital of the Jimma zone. The average annual rainfall ranges from 1400 to 1900 mm. The average annual minimum and maximum temperatures are 7°C and 31°C (Alemu et al. 2011).

### 2.2 Study design and sampling procedure

A cross‐sectional study design was employed for this study. The five study towns were selected purposively based on their high dairy production potential. The target population was smallholder dairy farmers in the five towns keeping indigenous zebu, crossbred, or both breeds of dairy cattle. A list of dairy‐keeping households in the area was obtained from the Livestock and Fisheries Resources Development Agency Offices (LFRDAO) of the respective districts. Accordingly, 151 smallholder dairy cattle owners from the five study towns were listed as a sampling frame. From this list, a total of 52 smallholder dairy‐keeping households (18 in Agaro, 6 in Yebu, 4 in Sheki, 12 in Serbo, and 12 in Seka) were randomly selected proportionally (proportional to size) to each districts’ sampling frame (Table 1) using a simple random sampling. Before the interview, selected dairy farmers were briefed on the purpose of the study, assured that their participation is voluntary and confidentiality of all information to be provided, and each respondent verbally gave informed consent to participate in the study.

**TABLE 1:** Proportionate distribution of urban farmers according to district towns

Town	Estimated number of dairy farmers	Proportion	Number selected
Agaro	52	(52/151)×52	18
Yebu	18	(18/151)×52	6
Sheki	12	(12/151)×52	4
Serbo	35	(34/151)×52	12
Seka	34	(34/151)×52	12
Total	151		52

### 2.3 Data collection

All data used in study were collected from a total of 52 smallholder dairy farming households by using structured questionnaire face‐to‐face interview survey. The questionnaire was based on open and closed questions. The questionnaire was developed in English and translated by the author into and conducted in the local language (Afaan Oromo). The translated questionnaire was pretested with smallholder dairy farmers who were not included in the final study. The pretested questionnaires were reviewed and modified accordingly for the actual data collection. Those questions which were not clear to the farmers were restructured and restated. To avoid bias between interviews and to validate the accuracy of the information, the questionnaires were administered by the first author who spoke the language of respondents, with support from staff of veterinary and livestock production of the respective districts. The questionnaire was used to collect information on household demographic characteristics (Belay, 2020), total daily milk production per household, daily milk used for home consumption, processing, and marketing; ranking of respondents on daily milk production, consumption of milk and milk products, form of milk consumption, traditional milk products processing, seasonal variation in milk consumption, daily per capita milk consumption, practice of butter, cheese, and ghee making; milk collection, storage, and processing equipment, milk fermentation time before processing, frequency of processing milk into butter and the amount of milk processed at one time, types of spices (plants) used during ghee making, milk marketing systems and channels, price of milk, contribution of income from milk to the total household income, means of milk transportation, and constraints of milk marketing.

### 2.4 Milk sample collection and analysis

Raw cow milk samples were collected aseptically from morning pooled milk container from 16 households (six from crossbred and ten from local dairy breed farms) who were being surveyed, and approximately 100 ml milk was collected and pooled for each breed of cows as per the procedure described by O’Connor (1995), in sterile containers and after thorough mixing. The samples were transported on icebox to Jimma University College of Agriculture and Veterinary Medicine, Animal Nutrition Laboratory, where they were analyzed on the same day. The raw milk samples were analysed separately for crossbred and local cows in duplicate using a rapid milk automatic milk analyzer ekomilk analyzer (MILCOSCOPE, Julie Z7 Scope Electric, Razgrad, Bulgaria) to determine the percentages of fat, lactose, protein, solid‐ not‐fat). Total solids were calculated by summing all milk solids. Determination of ash content (mineral contents) in raw cow milk was done according to the method of the Association of Official Analytical Chemistry (1990).

### 2.5 Statistical analyses

The data were coded and analyzed using SPSS version 20.0 (IBM, USA) software was used for all the statistical analyses to compute descriptive statistics for the variables. Descriptive statistics, such as means, percentages, and standard error of the means, were used to present the results. The Chi‐square (χ^2^) test was used to compare proportions of categorical variables among the towns. A *t*‐test was used to compare the raw milk chemical composition between the local and crossbred cows, and means differences were considered significant at *p* < .05.

## 3 RESULTS AND DISCUSSION

### 3.1 Milk production and utilization

Table 2 shows average daily milk production and utilization. The results revealed that on average 311 L of milk was produced by all respondents per day, excluding the milk suckled by calves. According to the farmers, there is seasonal variability in milk production, with more milk produced during the rainy season, attributed to adequate feed availability. Out of the total milk produced per day, about 195.5 L (62.9%) was for sale, 74 L (23.8%) was for home consumption, and 41.5 L (13.3%) was retained for processing at household level. This shows that a higher proportion of the milk produced was used for sale to generate cash income. This result is in agreement with the findings of Nigussie (2006), Sintayehu et al. (2008), and Yitaye et al. (2009), who reported that 79%, 74.2%, and 68% of the milk produced by urban dairy producers in their study was for sale, respectively.

**TABLE 2:** Mean (±*SE*) of milk produced, consumed, sold and retained for processing in litres/day/household in cross‐sectional survey of 52 dairy farmers in selected towns of Jimma zone, Ethiopia

Variables	Towns						*p*‐Value
	Agaro (*n* = 18)	Yebu (*n* = 6)	Sheki (*n* = 4)	Serbo (*n* = 12)	Seka (*n* = 12)	Overall (*n* = 52)	
Average milk production/d/household	5.03 ± 0.91^a^	9.17 ± 7.17^b^	10.25 ± 2.78^b^	5.75 ± 1.78^a^	4.62 ± 1.32^a^	5.98 ± 1.01	.531
Average milk retained for consumption/d (L)	1.30 ± 0.29^a^	1.25 ± 0.75^a^	3.25 ± 1.11^b^	1.50 ± 0.39^a^	1.00 ± 0.26^a^	1.42 ± 0.20	.086
Average milk retained for processing/d (L)	0.28 ± 0.13^a^	0.58 ± 0.30^a^	2.75 ± 1.11^b^	0.92 ± 0.35^a^	0.92 ± 0.27^a^	0.80 ± 0.16	.002
Average milk sold/d (L)	3.44 ± 0.78	7.33 ± 6.54	4.25 ± 2.66	3.33 ± 1.64	2.71 ± 1.18	3.76 ± 0.91	.713

Note

*n *= number of respondents; means with different superscript letters in the same row are significantly different at *p* < .05.

Abbreviations: d, day; L, litres.

The average milk production was 5.03 ± 0.91 liters/household/day. This finding is lower than the value of 27.12 L (Sintayehu et al., 2008), 43.0 L (Yitaye et al., 2009), and 10.21 to 15.90 L (Sintayehu et al., 2008) produced by smallholder urban dairy farmers elsewhere in Ethiopia. This difference could be attributed to the breed and number of cows, feeding regime, and general management practices. Average milk production per household in Yebu and Sheki (9.17 ± 7.17 and 10.25 ± 2.78) was significantly (*p* <.05) higher compared to the other towns. The amount of milk (liters) retained for home consumption and processing per day was significantly (*p* < .05) higher for Sheki compared to the other towns. The low milk production per household observed in this study could be attributed to the low productivity of indigenous cows and feed shortage. The majority of surveyed farmers in the present study kept small number of indigenous zebu cows, which are characterized by low milk production, and were relied on poor quality natural pastures as the main source of feed. Thus, upgrading of the local breed of cows through crossbreeding with exotic dairy genotypes and supplementation with concentrate feeds could lead to increased milk production. The respondents stated that milk production was fluctuated by season, where high milk yield was obtained during the rainy season due to better natural pasture availability compared to the long dry season.

### 3.2 Ranking of respondents based on daily milk production

Table 3 presents the ranking of respondents based on average daily milk production in the study area. There was a variation in the amount of milk produced and number of cows milked per day per household, with a minimum of one lactating cow being milked during the interview. A majority (59.6%) of respondents produced less than 5 L of milk per day, followed by 5–10 L (26.8%) and more than 10 L (13.6%) daily. This finding is not in agreement with the report of Tebug et al. (2012) who reported that majority (37%) of smallholder dairy farmers in Malawi produced 5–10 L of milk/day followed by <5 L (33%), 11–15 L (19%), and >15 L (11%). The variation in the amount of daily milk production among the farmers in the present study could be attributed to the difference in number and breed of lactating cows. The low genetic potential of local cows, unavailability of concentrate feeds, and the shortage of quality pastures available for grazing were among the most important problems facing farmers to increase milk production. According to the respondents, a higher daily milk production was acquired during the wet season due to better availability of natural pastures.

**TABLE 3:** Ranking of respondents based on average milk production per household/day in cross‐sectional survey of 52 dairy farmers in selected towns of Jimma zone, Ethiopia

Milk production (litres)	Percentage of farmers	Town and number of respondents					
		Agaro	Yebu	Sheik	Serbo	Seka	Total
<5	59.6	10	5	1	7	8	31
5–10	26.8	7	0	1	3	3	14
>10	13.6	1	1	2	2	1	7
Total	100	18	6	4	12	12	52

### 3.3 Household consumption of milk and its products

Table 4 presents summary results on consumption of milk and milk products at household level. As mentioned earlier, 23.8% of the total milk produced per household/day was retained for home consumption. Almost all the respondents (99%) indicated consuming milk at home on a daily basis when lactating cows are available. The dairy products consumed were fresh whole milk (14.4% of respondents), fermented milk (6.1%), a cottage yogurt commonly called *itittuu* in the local Afan Oromo language, which is usually fermented from raw milk, both fresh and fermented milk (21.1%), and a combination of fresh milk, fermented milk, butter, and cottage cheese (51.7%) of interviewed farmers. Raw milk is traditionally fermented for three to five days at ambient temperature and consumed either with meals (bread, *injera,* and porridge) or alone. The fermented milk is also churned into butter (*dhadhaa*) and consumed by family and also used for cosmetic purpose (hair ointment) mainly by females and children.

**TABLE 4:** The percentage of farmers interviewed consuming milk and milk products in the study area (multiple responses possible, % of respondents in each town and overall)

Variable	Towns						*p*‐Value
	Agaro (*n* = 18)	Yebu *n* = 6)	Sheki (*n* = 4)	Serbo (*n* = 12)	Seka (*n* = 12)	Overall (*n* = 52)	
Do you and your family consume milk and its products at home?							
Yes	94.4	100	100	100	100	98.9	.749
No	5.6	0	0	0	0	1.1	
Types of dairy product consumed?							
Fresh milk	38.9	16.7	0	16.7	0	14.4	.220
Fermented milk (*itittuu*)	5.5	16.7	0	0	8.3	6.1	
Fresh and fermented milk	38.9	33.3	0	16.7	16.7	21.1	
Fermented milk, cottage cheese and whey	0	0	0	8.3	0	1.7	
Fresh milk, fermented milk, butter and cheese	16.7	33.3	100	41.7	66.7	51.7	
Cheese and butter	0	0	0	8.3	0	1.7	
Fresh milk and all milk products	0	0	0	8.3	8.3	3.3	
Form of milk consumption							
Raw	5.5	16.7	0	8.3	0	6.1	.133
Boiled	94.4	66.7	50	83.3	66.7	72.2	
Both raw and boiled	0	16.7	50	8.3	33.3	21.7	
Is there seasonal variation in consumption of milk and milk products?							
Yes	50	50	50	50	66.7	53.3	.905
No	50	50	50	50	33.3	46.7	
Time of low or no milk and milk products consumption?							
Orthodox fasting periods	38.9	50	0	33.3	50	34.4	.686
Dry season	55.5	50	100	66.7	50	64.4	
Wet season	5.5	0	0	0	0	1.1	

In this study, majority (72.2%) of respondents consumed boiled milk, followed by both boiled and raw milk (21.7%) and raw milk (6.1%). The practice of raw milk consumption could lead to increased risk of milk‐borne illness in consumers and needs attention. Thus, there is a need to increase awareness of the farmers on risks of raw milk consumption and advise them to boil their milk before consumption to protect their health and that of their family. Moreover, appropriate risk‐management strategies need to be implemented to protect particularly children who are highly susceptible to milk‐borne diseases and for whom milk is a beneficial dietary component. In rural Ethiopia, farmers prefer consumption of raw fresh milk and raw fermented milk, and they perceive that boiling milk reduces its flavor and taste of milk. More than half (53.3%) of respondents reported the existence of seasonal variation in milk consumption. About 64.4% and 34.4% of respondents reported that household milk consumption decreases during the dry season and major Orthodox Christians’ fasting periods (Christmas and Easter). The longest fasting periods are before Christmas (40 days) and Easter (55 days), and in August (16 days). During these fasting periods, majority of Orthodox Christians abstain from consuming animal source food including dairy products, except children (below 7 years old) who are not imposed to fast. This is the time when milk sales and prices and consumption drop compared to non‐fasting periods. In addition to the main fasting periods, majority of Orthodox Christians practice fasting on Wednesday and Friday all year round, except the two months after Easter. Knutsson and Selinus (1970) reported that although fasting rules are strict, lactating and pregnant women, severely ill or weak persons, as well as children below the age of seven can be fully excused from fasting. The main reason mentioned for decreased milk consumption during the dry season was associated with low milk production due to feed shortage in terms of quality and quantity.

### 3.4 Per capita consumption of milk in households

Traditionally, milk plays an important part in daily nutrition of the surveyed households. As indicated in Table 5, the overall average daily milk consumption per capita was 0.148 L per household member of the interviewed respondents, and this corresponds to average per capita consumption of about 73.84 L per year, which is over three times higher than the national average per capita consumption of 19 L per year. Even though the average per capita/day milk consumption was 148 ml (0.148 L), it could vary from household to household based on the amount of milk produced, number of lactating cows, family size, and number of young children per household. Usually, adult family members seem to consume less milk compared to children and sick family members. Households with large number of lactating cows tend to consume more milk compared to those with less number of lactating cows.

**TABLE 5:** Average per capita per day consumption of milk by household members of respondents during the survey in the study area

Parameters	Daily per capita milk consumption^a^
Number of respondents	52
Total family size of the respondents	305
Average milk consumed/all households/day, kg	73.84
Estimated per capita consumption of milk/day, kg	0.242
Per capita milk consumption per year, kg	88.33

^a^ Average milk consumption per capita/day = milk retained for home consumption/household/day in kg divided by the total family size of all the respondents.

### 3.5 Traditional processing of milk into butter, cheese, and ghee

The study shows that some of the respondents reported practicing traditional milk processing at the household level into fermented milk, butter, cottage cheese, and ghee. Of all the respondents, 42.2% practiced converting fermented or sour milk into butter. About 41.1% of respondents fermented whole milk for three days at ambient temperature before processing into butter. The farmers said that milk fermentation time was shorter during the dry season due to high ambient temperature compared to rainy season. Majority (41.1%) of respondents reported processing fermented milk into butter once a week. About 38.9% of respondents processed on average 5.0 L of fermented milk into butter at a time. These findings are in agreement with that reported by Shiferaw et al. (2003) who stated that 61.8% of dairy farmers in their study did not process milk due to low milk production. The primary milk processing products produced by the interviewed households were butter, cottage cheese, ghee, and fermented whole milk. Majority (22.7%) of respondents reported processing milk into butter, cheese, ghee, and fermented milk. Majority (38.9%) of respondents churned on average 5 L of milk at a time. This finding is slightly lower than the average of 6 and 6.4 L of milk processed into butter at a time as reported by Abebe et al. (2013) and Zelalem and Ledin (1999), respectively (Table 6).

**TABLE 6:** Types of traditional milk processing products (%) as reported by 22 respondents who involved in processing milk in the study area

Variable	Towns						*p*‐Value
	Agaro (*n* = 18)	Yebu *n* = 6)	Sheki (*n* = 4)	Serbo (*n* = 12)	Seka (*n* = 12)	Overall (*n* = 52)	
Do you process milk at home?							
Yes	11.1	0	75	58.3	66.7	42.2	.001
No	88.9	100	25	41.7	33.3	57.8	
Frequency of processing milk							
Every week	5.5	0	75	58.3	66.7	41.1	.039
Occasionally	5.5	0	0	0	0	1.1	
Major milk processing products^a^							
Cottage cheese (ayib)	0	0	0	0	8.3	1.7	.000
Butter, cottage cheese and ghee	0	0	0	41.7	50	18.3	
Cheese, fermented milk, butter, ghee	0	0	50	25	25	20.0	
Cheese, fermented milk, butter	22.2	33.3	50	8.3	0	22.7	
Cheese and butter	0	16.7	0	0	0	3.3	
Butter milk	11.1	0	75	58.3	66.7	42.2	
When do you processed milk frequently							
Fasting periods							
Wet season							
Duration of milk fermentation before processing							
Three days	11.1	0	75	58.3	66.7	41.1	
Five days	5.5	0	0	0	0	1.1	
The average amount of milk processed at a time							
Five liters	11.1	0	75	41.7	66.7	38.9	.002
Seven liters	0	0	0	16.7	0	3.3	

^a^ Only for households who process milk to different milk products.

#### 3.5.1 Traditional butter making

For butter making, milk is collected over a period of three to five days in a clay pot or plastic can or gourd and allowed to naturally fermented for three to five days based on the season. When the milk has fermented and sufficient milk has been collected, it is poured into clay pot or gourd and shaken back and forth for about one to one and half hours, depending on the quantity of fermented milk, temperature, acidity of milk, and person churning until butter granules are formed. According to the respondents, during butter making, the breakpoint of butter grain formation is known through a change in the sound made while churning. When butter granules are formed, the churn is opened; the butter is separated and skimmed off. Then it is kneaded and washed in clean cold water. These results are in agreement with the observations of Eyassu and Asaminew (2014).

#### 3.5.2 Traditional ghee making

The traditionally produced butter was later refined to get traditional ghee (clarified butter oil) to increase its shelf life. Ghee was made by first washing the butter to remove any impurities and heating butter on open fire using either wide‐mouthed clay pot or metal dish in order to remove the water content by melting. Heating and stirring continues until foam is formed and a clear liquid is obtained. Along heating the butter, combination of spices are added to induce good aroma, increased shelf life, and taste. Heating of melted butter is continued until bubbling ceases and all moisture evaporates (assumed that foam and bubble are appearing due to water evaporation). Melted butter is then filtered through sieve or piece of cheesecloth to remove impurities and decanted into another vessel leaving the curd material in the dish. Well‐dried containers free from moisture with tight stopper are used to keep refined butter either for use or for future preservation. Small amount is daily removed and used in cooking and preparation of various traditional foods (Almaz et al., 2001; Tola & Beyene, 2012). Similar procedures of ghee making have been reported in previous studies (Alganesh, 2002; Asaminew, 2007; Debela et al., 2016; Eyassu & Asaminew, 2014) elsewhere in Ethiopia. Ghee has a good keeping quality than butter which allows storage for more than a year without significant deterioration (Almaz et al., 2001; Eyassu & Asaminew, 2014).

All the respondents (100%) stated that the butter was produced at household level for self‐consumption only, after converting it into traditional ghee. The ghee is used for making the traditional stew (*Wot*) that is eaten with bread‐like Injera made from Tef (*Eragrostis tef*), maize, wheat, and other cereals. The respondents indicated that the ghee produced was not for sale because it was just enough for family consumption only. In addition to home consumption, butter was used for cosmetics (hairdressing by women and children).

#### 3.5.3 Cheese making

For making cottage cheese, the buttermilk (by‐product of butter making) is heated in clay or metal vessel on fire, generally until sufficient coagulation of casein was reached. When adequate casein is coagulated, the pot is removed from the fire and allowed to cool for some time. Then the cheese is collected in a clean container after draining off as much whey as possible from the coagulate. According to the respondents, the moisture content of the cheese affects its keeping quality so that the whey should be removed from the cheese completely. All the respondents reported they keep all the cheese only for home consumption. The produced cheese is either consumed as it is or mixed with butter, salt, and spices before consumption with meals. The procedures of cottage cheese making reported in the present study support the observations of previous studies (Alganesh, 2002; Eyassu & Asaminew, 2014). The time taken for cheese making was reported to vary based on the amount of buttermilk used, type of container, and fire intensity during buttermilk cooking.

### 3.6 Milk storage and processing containers

Gourd (*Lagenaria siceraria;* 36.1%), clay pot (3.3%), and plastic jar can (2.8%) were the main containers used for milking and milk storage. It has been reported that in East Wollega, 91% of women used gourd for churning and storage of milk (Alganesh, 2002). The results also showed that 37.8% and 4.4% of farmers used plastic jar can and clay pot for processing fermented milk into butter. The fermented milk is placed in a clay pot or plastic jar can and shaken or agitated until the butter grain will form lumps of butter, which is known by the change in the sound of the milk being churned. Then, the butter is skimmed off, kneaded in cold water, and washed to remove any remaining buttermilk. Belay and Janssens (2014) reported that 80% of small‐scale dairy producers in Jimma town used plastic jar can for butter making. The use of clay pot for butter making observed in the present study is similar with the report of Yilma and Inger (2001) in the central highlands of Ethiopia. The finding of the present study is not in agreement with the observations of Sintayehu et al. (2008) and Alganesh (2002) who indicated that 96.5% and 91% of the dairy farmers used clay pot and gourd for churning, respectively, in southern and western Ethiopia (Table 7).

**TABLE 7:** Milk storage and processing materials as reported by 22 respondents who process milk at home in the study area

Variable	Study towns						*p*‐value
	Agaro	Yebu	Sheki	Serbo	Seka	Overall	
Materials used for making butter							
Traditional clay pot	5.5	0	0	16.7	0	4.4	.020
Bottle gourd/calabash	5.5	0	75	41.7	66.7	37.8	
Milk storage containers							
Clay pot	0	0	0	16.7	0	3.3	.081
Gourd/calabash	5.5	0	75	41.7	58.3	36.1	
Plastic jar can	5.5	0	0	0	8.3	2.8	

### 3.7 Spices used in traditional ghee making

The results show that *Allium cepa, Aframomum angustifolium, Allium ursinum, Cordiandrum sativum, Nigella sativa, Ocimum sanctum, Satujera species, Trachyspermum copticum, Zingiber officinale*, etc. were the most commonly used spices or plants to impart good flavor and taste to the ghee, and to preserve it for longer periods without spoilage. The spices used during the ghee making varied from household to household, and coriander and turmeric were reported to be the less frequently used spices during ghee making (Table 8).

**TABLE 8:** Spices (plants) used in the traditional ghee making as reported by respondents (spouses) in the study area

Vernacular name	Common name	Scientific name	Plant parts used
Dimbilaala^a^	Coriander	*Coriandrum sativum*	*Seeds, stems and leaves*
Habasuuda adii	Bishop's weed	*Trachyspermum ammi (L.)*	*Seeds*
Habasuuda gurraacha	Black cumin	*Nigella sativa*	*Seeds*
Irdii^a^	Turmeric	*Curcuma domestica*	*Tuber*
Jinjibila	Ginger	*Zingiber officinale*	Tuber
Kusaayee	Lantana	*Lantana trifolia*	Leaves
Oogiyoo	Kororima	*Aframomum corrorima*	Seeds
Qullubbii adii	Garlic	*Allium sativum*	Tuber
Siqaaqibee	Basil	*Ocimum spp*	Seeds, stems and leaves
Sunqoo	Fenugreek	*Trigonella foenum‐graecum*	Seeds
Xoosinyii	Oregano	*Origanum vulgare*	Leaves

^a^ Less frequently used spices.

### 3.8 Types of milk products marketed

Marketing is a very important aspect of the dairy chain. Presence of close‐by markets for milk and dairy products is a key motivating factor for milk producers. Nearly more than half of the respondents reported selling dairy products while 47.7% of them in the study area did not market any milk at the time of the interviewing. The results of the current study showed fresh whole milk (12.7%) was the main dairy product sold, followed by cottage cheese (1.9%) and traditional butter (1.5%) (Table 9).

**TABLE 9:** Types of milk and traditional milk products sold by the respondents in the study area

Variables	Towns						*p*‐Value
	Agaro	Yebu	Sheki	Serbo	Seka	Overall	
Do you sell milk and milk products							
Yes	92.3	42.4	17.2	46	63.4	52.3	
No	6.7	57.6	82.8	54	36.6	47.7	
Types of milk and milk products sold							
Fresh raw milk	28.8	5.8	3.8	11.5	13.5	12.7	.296
Cottage cheese (*ayib*)	0	5.8	0	0	3.8	1.9	.004
Butter	0	5.8	0	0	1.9	1.5	.001
Frequency of selling raw milk							
Every day	28.8	7.7	1.9	9.6	15.4	12.7	.057
Every other day	0	0	1.9	0	0	0.4	
During fasting period only	0	0	0	1.9	1.9	0.8	
Frequency of selling *ayib* and butter							
Once per week	0	5.8	1.9	0	3.8	2.3	.009
Reasons for selling milk							
Source of cash income	21.2	1.9	7.7	19.2	23.1	14.6	.000
To buy household necessities	0	5.8	0	0	0	1.2	
Both income and household necessities	13.5	3.8	0	3.8	0	4.2	

### 3.9 Means of milk transportation

As indicated in Table 10, majority (53.8%) of respondents indicated that they sold milk directly to consumers and retailers at farm gate (point of production), whereas 36.5% reported that they delivered milk to customers’ houses or place of business (cafes, hotels, restaurants) by family members or hired laborers. Transporting the milk from farm to their customers was mainly done on foot (96.2%).

**TABLE 10:** Means of milk delivery and transportation

Variables	Towns						*p*‐Value
	Agaro (*n* = 18)	Yebu (*n* = 6)	Sheki (*n* = 4)	Serbo (*n* = 12)	Seka (*n* = 12)	Overall (*n* = 52)	
Means of milk delivery to customers							
Collected by consumers and retailers at farm gate	22.2	33.3	100	58.3	91.7	61.1	.025
Family or hired labour	61.1	100	0	41.7	0	40.6	
A combination of the above	16.7	0	0	0	8.3	5.0	
Means of milk transportation to customers							
Foot	94.4	83.3	100	100	100	95.6	.416
Vehicle	5.5	16.7	0	0	0	4.4	

### 3.10 Milk marketing systems and channels

A market can be visualized as a process in which ownership of goods is transferred from sellers to buyers who may be final consumers or intermediaries (Debrah & Berhanu, 1991). In the present study, respondents sold their morning and evening milk immediately after milking to customers (consumers and retailers). The study revealed that raw milk was sold through informal channel (100%) without any quality supervision. It is the direct sale of milk to neighbors (consumers) or retailers (cafes, hotels, institutions, restaurants, and tea houses). The problem of this system is the lack of milk quality control due to consumers’ low awareness of food quality and safety, and lack of standards that maintain milk safety, quality and food security, and animal welfare regulation (feeding, health, housing, sanitation, etc.) standards. Thus, the consumption of raw milk may cause diseases that threaten health through milk‐borne infectious diseases. Moreover, the farmers did not respect veterinary drug withdrawal period after treatment with antibiotics due to limited knowledge of potential human health effects of the drug residues and poor extension services. Thus, this calls for the need of training and awareness creation of farmers on effects caused by antibiotic residues in milk (Table 11).

**TABLE 11:** Milk marketing system and channels as reported by 52 respondents in the study area

Variables	Towns						*p*‐Value
	Agaro (*n* = 18)	Yebu (*n* = 6)	Sheki (*n* = 4)	Serbo (*n* = 12)	Seka (*n* = 12)	Overall (*n* = 52)	
Milk marketing system							
Informal	100	100	100	100	100	100	
Milk marketing channels							
Producer →Consumers	22.2	83.3	100	66.7	100	74.4	.002
Both Producer →Consumers and Producer →Retailers →Consumers	77.8	16.7	0	33.3	0	25.6	
Do you experience milk marketing problem?							
Yes	11.1	16.7	25	41.7	16.7	22.0	.358
No	89.9	83.3	75	58.3	83.3	78.0	
Milk marketing problems							
Low price and price fluctuations	100	100	100	100	100	100	
Primary criteria for selection of selling outlets							
Fair price	16.7	33.3	75	50	25	40	.120
Sustainable contract	83.3	66.7	25	50	75	60	
Is there a period of low demand for milk							
Yes	11.1	16.7	25	41.7	16.7	22.2	
No	89.9	83.3	75	58.3	83.3	70.0	
Period of low demand for milk							
Fasting periods	11.1	16.7	25	41.7	16.7	22.2	.358

When selling milk, farmers received the full price paid by their customers based on the volume of milk supplied with no quality (chemical composition) and hygienic (bacteriological) control, and payments were collected once either in advance or at the end of the month, based on verbal agreement made between producer and customers. The findings of the present study are in agreement with the reports of earlier studies (Geleti et al., 2014; Sintayehu et al., 2008; Yitaye et al., 2009) who also observed informal marketing of milk elsewhere in Ethiopia. Gebreegziabher and Tadesse (2014) reported that about 80% of the milk sold in Kenya goes through the informal channels.

The main milk marketing channels practiced by respondents in the stud area were Producer →Consumers (63.5%) and both Producer →Consumer and Producer →Retailers (cafes, hotels, restaurants, institutions) →consumers (35.5%) without any quality evaluation. Yitaye (2008) reported that direct delivery to nearby consumers was the primary milk outlet for producers, followed by retailers for the urban and peri‐urban systems, respectively. Retailers, in the context of this study, include hotels, restaurants, coffee and tea houses, and cafeterias. The milk marketing system in the study area was characterized by no license to operate, low cost of operation, high producer prices as compared with formal market, and no regulation of operation (SNV, 2008). In smallholder dairy farming, 80% of the milk marketed passes through the traditional channels handling raw milk and traditional processed products (Kumar & Staal, 2010; Staal et al., 2006). Long‐term contractual arrangements with buyers (67.3%) and better price (32.7%) were farmers’ preferred milk marketing outlets. The major constraints of milk marketing were low milk production of indigenous cows, low prices of milk, seasonal fluctuation in milk production, and lack of dairy cooperatives and milk collection centers. One of the reasons for the low prices of milk was the decreased demand for dairy products during fasting periods of Orthodox Christians, especially for one month before Christmas and 2 months before Easter. From results of this study, it is suggested that the formation of farmers’ milk marketing groups or dairy cooperatives could be helpful in deciding milk price, having better access to formal credits, to obtain external support or trainings from the government and private sectors, and credit arrangements could be easily organized within the group.

### 3.11 Price of milk

The study revealed that dairy farmers in the study area sell milk directly to consumers or retailers (cafes, hotels, tea houses, and restaurants). The average price of milk per liter was 5.0 Ethiopian Birr or 0.053 US Dollar at the time of this study. The farmers stated that they received low price for milk, which less motivated them to improve milk production. Milk is sold on contractual basis and payments are collected mainly at the beginning or end of a month based on the agreements with customers (Table 12).

**TABLE 12:** Price of milk per kg in Ethiopian Birr (ETB) in the study area

Town	Distance			
	*n*	Mean ± *SE*	Minimum	Maximum
Agaro	18	4.83 ± 0.18^a^	4.00	6.00
Yebu	6	5.00 ± 0.52^a^	3.00	6.00
Sheki	4	5.50 ± 0.29^a^	5.00	6.00
Serbo	12	5.08 ± 0.31^a^	3.00	6.00
Seka	12	4.62 ± 0.16^a^	3.00	6.00
Overall	52	4.91 ± 0.12	3.00	6.00
*p*‐value		.449	3.00	6.00

Note

Means with different superscript letters in the same column are significantly different at *p* < .05.

### 3.12 Farmers’ perceived contribution of milk to household income

Majority (45%) of respondents indicated that income from milk contributed about 10% of the household income. About 18.3%, 10%, 9.4%, 6.7%, and 6.7% of respondents reported that the contribution of the sales of milk to the household income was 40%, 50%, 30%, 75%, and 95%, respectively. The differences in income from sale of milk could be attributed to the number of milking cows, breed of cows, and the amount of milk produced per household (Table 13).

**TABLE 13:** Contribution of milk selling to the household income according to the respondents in the study area (% of respondents in each town and overall)

Proportion of dairy income to gross household income (%)^a^	Towns						
	Agaro	Yebu	Sheki	Serbo	Seka	Overall	*p*‐Value
10	0	0	0	16.7	8.3	5	.183
20	2.2	33.3	100	16.7	50	45	
30	5.5	0	0	33.3	8.3	9.4	
40	16.7	33.3	0	25	16.7	18.3	
50	16.7	16.7	0	8.3	8.3	10	
60	5.5	0	0	0	0	1.1	
75	16.7	0	0	0	8.3	6.7	
95	16.7	16.7	0	0	0	6.7	

^a^ Proportion of income estimates were based on the assessment of the respondents

### 3.13 Chemical composition of raw cow milk

The effect of cow genotype on milk ash, fat, lactose, protein, solids‐not‐fat (SNF), and total solids (TS) content is presented in Table 10. There was a significant effect (*p* < .05) of genotypes on milk fat, lactose, and protein. However, there was no significant difference (*p* < .05) in the composition of ash, SNF, and TS between the two different dairy cattle genotypes. The results show that the mean ash, fat, lactose, protein, SNF, and TS content of milk of the crossbred cows were 0.73 ± 0.01, 3.97 ± 0.01, 5.66 ± 0.01, 3.28 ± 0.03, 8.55 ± 0.02, and 12.53 ± 0.37, respectively. These findings are in close agreement with the findings of Asaminew (2007) who reported 4.14, 3.45, 13.15, 0.70, and 8.96% fat, protein, total solids, ash, and SNF contents for crossbred cows’ milk, respectively (Table 14).

**TABLE 14:** The effect of cow genotype on raw milk chemical composition in the study area

Chemical composition	Breed of cow			*p*‐Value
	Crossbreed mean (± *SD*)	Local mean (±*SD*)	Overall mean Mean (±*SD*)	
Ash (%)	0.73 ± 0.01	0.72 ± 0.01	0.72 ± 0.00	.312
Fat (%)	3.97 ± 0.01^a^	4.45 ± 0.01^b^	4.21 ± 0.24	.000
Lactose (%)	5.66 ± 0.01^a^	5.43 ± 0.01^b^	5.54 ± 0.11	.001
Protein (%)	3.28 ± 0.03^a^	3.11 ± 0.01^b^	3.19 ± 0.8	.033
Solids‐not‐ fat (%)	8.55 ± 0.02	8.46 ± 0.37	8.50 ± 0.40	.822
Total solids (%)	12.53 ± 0.02	12.91 ± 0.37	12.72 ± 0.19	.413

Note

Mean (±*SD*) values within rows with different superscript letters differ significantly at *p* < .05.

Abbreviation: *SD*, standard deviation.

The results indicated that the mean ash, fat, lactose, protein, SNF, and TS content in milk of the indigenous cows were 0.72 ± 0.01, 4.45 ± 0.01, 5.43 ± 0.01, 3.11 ± 0.01, 8.46 ± 0.37, and 12.91 ± 0.37%, respectively. This finding concur with the findings of Asaminew (2007) who also reported values of 4.71, 3.25, 13.47, 0.73 and 8.78 for fat, protein, total solids, ash and solids‐not‐fat (SNF) contents of raw milk for local cows, respectively. The minimum fat percent for whole cow milk recommended by the Ethiopian Standards Agency (ESA) should not be less than 3.5% (ESA, 2009). The SNF content of raw milk of both breeds of cows in the present study is higher than the minimum standard (8.25%) for SNF content of whole cow milk (FDA, 2010). The TS content of milk found in the present study is slightly higher than the minimum standards for TS content of cow milk established by the European Union, which should be not <12.5% (FAO, 2000). The overall mean protein content of raw milk reported in the current study is slightly lower than the minimum of 3.2% recommended by the ESA (2009). The low protein content of the milk in the present study could be due to the low protein contents of natural pasture, the major source of dairy cattle feed in the area, and lack of supplementary feeding with protein‐rich concentrates. Generally, milk composition can be very variable depending on many factors such as: breed and the health condition of the animals, lactation period, feeding management (type and quality), season, method of milking (manual or automatic), age and the number of lactation, individual cows and environmental factors (Pandey & Voskuil, 2011; Wolfson & Sumner, 1993). Respondents used color, smell, and taste as criteria to evaluate milk quality.

According to the ESA, the minimum fat percent for whole milk should not be less than 3.5 percent (ESA, 2009). Hence, the average fat percent in the current study fulfills the recommended range even though it is below average for the local breeds. According to the ESA, the minimum percent protein content of whole milk should be 3.20 percent (ESA, 2009). Hence, the average protein content for the current study is slightly below the recommended standard for the nation.

According to the standards set by the ESA, the minimum average percent total solids content of unprocessed whole cow milk should not be less than 12.8 percent.

The overall average of lactose content in this study showed 5.54 ± 0.11 percent. According to the European Union Quality standards for unprocessed whole milk, the lactose content should not be less than 4.2 percent (Tamime, 2009). The minimum SNF percent set by European Quality Standards for unprocessed whole milk is 8.5 percent (Tamime, 2009).

### 3.14 Dairy farmers’ awareness of cattle‐ and milk‐borne zoonotic diseases

Overall, 100% and 80% of the respondents had awareness (knowledge) about at least one cattle‐ and milk‐borne zoonotic disease. About 66.7%, 15%, 6.1%, and 79.4% of respondents knew that anthrax, bovine tuberculosis, mastitis, and taeniasis are cattle‐borne zoonotic diseases transmitted to humans through consumption of raw meat of infected animals and contact with infected animals. Whereas 6.1%, 6.1%, 20%, and 6.1% respondents knew that anthrax, brucellosis, bovine tuberculosis, and mastitis are milk‐borne zoonotic diseases transmitted to humans through consumption of raw milk from infected animals. Girma et al. (2012) reported that the main zoonotic diseases mentioned by surveyed farmers in their study were rabies (100%), anthrax (94.27 %), taeniasis (89.06 %), tuberculosis (88.54%), brucellosis (49.48%) and infectious diseases of zoonotic importance (31.25 %).

In this study, respondents had a relatively lower level of awareness about zoonotic diseases. This study is in agreement with the findings of Amenu et al. (2010) who reported that a high number of farmers had no thorough and accurate awareness about zoonotic diseases. In contrast, Girma et al. (2012) reported higher level of zoonotic awareness of respondents in Addis Ababa. The low level of awareness about zoonotic diseases in the present study could be due to poor communication between veterinarian and human healthcare professionals about zoonotic diseases. This low level of awareness and knowledge would likely expose dairy farmers and consumers to increased risk of zoonotic diseases. Hence creation of awareness among dairy farmers is of paramount importance in control of zoonotic diseases to improve animal health, productivity, food safety, and human health in the study area and for similar settings. The findings from this survey have the potential to inform policies aimed to enhance zoonotic disease control and to develop strategies to enhance dairy production. The results also provide the basis for key extension messages to improve health and productivity of dairy cattle and public health threats caused by zoonotic diseases. In this study, 100%, 100%, and 80% of respondents indicated that contact with infected animals, consumption of raw meat from infected animals, and consumption of raw milk from infected cows are the main routes of transmission of cattle‐ and milk‐borne zoonotic diseases, respectively (Table 15).

**TABLE 15:** Dairy producers’ awareness of cattle and milk‐borne zoonotic diseases among smallholdings in selected towns of Jimma zone, Ethiopia (% of respondents)

Variables	Towns					
	Agaro	Yebo	Sheki	Serbo	Seka	Overall
Are you aware of any one cattle zoonotic disease						
Yes	100	100	100	100	100	100
No	0	0	0	0	0	0
Known cattle borne zoonotic diseases^a^						
Anthrax	83.3	50	75	50	75	66.7
Brucellosis	0	0	0	0	0	0
Bovine Tuberculosis	5.5	0	0	8.3	0	2.8
Mastitis	5.5	0	0	25	0	6.1
Taeniasis	88.9	66.7	75	83.3	83.3	79.4
Are you aware of at least one milk‐borne zoonotic disease						
Yes	32.2	16.7	0	75	25	29.8
No	67.8	83.3	100	25	75	70.2
Milk borne zoonotic diseases^a^						
Anthrax	5.5	0	0	25	0	6.1
Brucellosis	5.5	0	0	25	0	6.1
Bovine Tuberculosis	16.7	16.7	0	25	25	20
Mastitis	5.5	0	0	25	0	6.1
Route of transmission of cattle and milk‐borne zoonotic diseases						
Contact with infected animals^a^	100	100	100	100	100	100
Consumption of infected meat^a^	100	100	100	100	100	100
Consumption of infected milk^b^	100	100	0	100	100	80

^a^ Multiple responses possible.

^b^ For those respondents who had awareness of cattle‐borne zoonotic diseases.

^c^ For those respondents who had awareness of milk‐ borne zoonotic diseases.

## 4 CONCLUSION

In the current study, milk chemical composition, processing of milk products, marketing, and consumption pattern of milk and milk products was assessed. The results showed that the average milk produced per household per day was low mainly due to the use of unimproved local breed of cows for milk production. Out of the total milk produced, the highest proportion was sold as raw milk through informal marketing system and with no quality control. Milk processing into butter is still traditional, which leads to inefficient fat recovery. Raw milk was channeled through producers to consumers and producers to retailers (cafes, hotels, and restaurants). The average price paid per volume of milk was low. In order to obtain better price, an efficient milk marketing strategy should be adopted. Hence, establishing of dairy cooperative or milk marketing group is essential for creating formal milk marketing linkage between producers and consumers or retailers. A total of 11 spices (plants) used in traditional ghee making to improve shelf life and taste were identified. The study revealed that fat, lactose, and protein contents of raw milk differed between crossbred and indigenous cow breeds. However, future studies could potentially complement the findings of this study by assessing large sample sizes in different seasons to compare how breed type affects milk nutritional quality in order to recommend the breed of cows farmers should keep to produce milk of high quality needed by consumers and processors. The awareness level of dairy farmers about cattle‐ and milk‐borne zoonotic diseases was found to be low due to lack of adequate information. Thus, there is a need to raise dairy farmers’ awareness on prevention and control of zoonotic diseases to reduce their potential spread and risks to human health. Moreover, there is an urgent need for awareness creation among milk producers and consumers to boil raw milk before consumption to mitigate contracting zoonotic diseases in the study area.

## CONFLICTS OF INTEREST

The author declares no conflict of interest.

## ETHICAL APPROVAL

No ethical approvals were required for the study. Verbal consent was obtained from each of the dairy keepers after explaining the purpose of the study prior to the start of data collection. Verbal consent was used because most of the livestock keepers do not know how to read and write. The procedure used in the study, in particular sample collection, did not harm humans or animals. Participation in this study was voluntary with strict confidentiality of information and freedom to stop participating at any time.

## Data Availability

The data that support the findings of this study are available on request from the corresponding author.
